# Influence of Bubbles Causing Cavitation on Spool Oscillation of a Direct Drive Servovalve

**DOI:** 10.3390/mi12060717

**Published:** 2021-06-18

**Authors:** Pengda Ren, Bin Wang, Wei Zhang, Zhigang Xie

**Affiliations:** 1 College of Energy and Power Engineering, Nanjing University of Aeronautics and Astronautics, Nanjing 210016, China; rpdwjr@nuaa.edu.cn; 2 Jiangsu Province Key Laboratory of Aerospace Power System, Nanjing University of Aeronautics and Astronautics, Nanjing 210016, China; 3 Flight Automatic Control Research Institute, Xi’an 710000, China; zhangwei908@163.com (W.Z.); xiefriend@gmail.com (Z.X.)

**Keywords:** direct drive servovalve, spool oscillation, cavitation, surface average cavitation, shedding bubbles

## Abstract

A direct drive servovalve has some inherent benefits over its conventional counterparts, but also has better reliability and output power. However, due to the rigid connection between the spool and the motor, which takes the place of interstage drive-by fluid, the spool oscillation is a long-standing unsolved problem. In order to study the oscillation mechanism and the influencing factors, a double-circuit direct drive servovalve was numerically simulated. An oil return valve cavity was concentrated on as the main flow domain and was used to analyze the fluid flow characteristics. Local cavitation fraction and surface average cavitation fraction were defined to evaluate the cavitation situation. The periodic growth process of bubbles in the valve cavity was obtained. The numerical results show that bubbles in the oil return valve cavity changes, although the occurrence, evolution, and collapse stages were certain. The intensity of pressure pulsation caused by bubble variation is highly related to the bubbles causing the cavitation, which suggests a workable way to inhibit the spool oscillation.

## 1. Introduction

The electro-hydraulic system has many advantages, including, but not limited to, a compact structure, high power density, fast response and a large rigidity. This is why it is extensively used in the aerospace field [1]. Generally, an electrohydraulic servo system involves a servovalve as a core component [2]. However, electrohydraulic servovalve failure is common in airborne control systems and threatens control performance and even flight safety seriously. For example, a servovalve fault may exhibit spool oscillation, squeal noise, motion catching, etc., which can make the performance of the control system worse and can have catastrophic consequences [3,4,5]. Much work has been done on failure diagnosis and on reliability improvements for servovalves. Therefore, direct drive servovalves are a promising alternative. They adopt a linear or rotary motor to move the spool instead of a pilot stage. This arrangement allows a much larger spool opening for stronger contamination endurance and a higher output power than conventional two-stage servovalves [6,7]. Unfortunately, the direct drive scheme avoids interstage fluid power transmission but introduces touch interaction. It usually transmits power by the mechanical connector between the motor and the spool [8]. A double-circuit valve serves as a control component in the double surface actuation system in aircraft. It often employs a long and thin link to push or pull the spool. Spool oscillation is a long-standing unsolved problem, which can be associated with long link stiffness, fluid-solid coupling or self-excitation [9,10].

The self-excited oscillation of a servovalve refers to the automatic vibration of movable components within the valve, which is usually associated with the hydraulic power source, and not external excitation. In general, a servovalve self-excited oscillation frequency can reach as high as hundreds to thousands of Hertz [11,12]. Previous studies show that the self-excited oscillation of a servovalve is closely related to transient cavitation and pressure pulsation in the flow field [13].

Elsheikh et al. [11] used simulation and experiments to study the high-frequency noise generated during the operation of a balance valve. The influence of different components involved in noise generation was investigated, and design modifications were introduced to eliminate the undesired effect, but without altering the operation envelope or performance of the valve. Ziada et al. [14] conducted another study on the oscillation noise of a relief valve and a turbine control valve. Both of these studies agreed that the shear layer may induce a pressure pulsation and high-frequency oscillation noise in a servovalve. Dianrong Gao et al. [15] found that there is a large eddy current in valve cavities, the shape and strength of which is affected by valve opening, as well as energy loss, noise, and steady fluid flow force in the flow field inside valves. Based on a numerical simulation of flow in servovalves, Li et al. [16] studied the reason for the cavitation, which was a change of eddy current in the flow field. This directly affected the energy loss and generation of fluid noise. Liu et al. [17] combined numerical and experimental investigation on unsteady cavitation flow and pressure fluctuation characteristics in regulating valves. They discovered that the changes in the length/radius ratio of a valve spool is an important factor affecting unsteady cavitation flow and pressure pulsation in the valve. The size of cavitation bubbles increased initially and then decreased over time for one cycle. With the increasing length/radius ratio, the oscillation cycle of the bubbles extends. Qiu et al. [18] analyzed the pressure drop, velocity, and vapor volume distribution in regulating valves. The total vapor volumes were predicted and analyzed. Their work found that a decrease in the valve core displacement induced the enlargement of the vapor distribution region and increased vapor density.

Some studies have preciously investigated direct drive servovalves [19,20,21]. However, due to high-pressure drop across small openings of the spool, the fluid is prone to separation or vaporization when flowing through the valve, which can affect the performance of the valve significantly [22,23].

The purpose of this study was to investigate the cavitation growth, evolution and the affecting mechanism on spool oscillation in a double-circuit direct drive servovalve. Two-phase numerical simulations were implemented under different operations to examine the effects of cavitation intensity on the oscillation. This work provides several effective ways to suppress the oscillation inside the valve.

## 2. Numerical Methods

### 2.1. Structure of the Servovalve

The double-circuit direct drive servovalve is composed of two identical three-way four-port valves, the spool of which are united as one by a rigid connection. A linear motor drives the spool to achieve the synchronous flow regulation of double-circuit hydraulic systems, by control of two-pair ports in the spool simultaneously.

**Figure 1:**
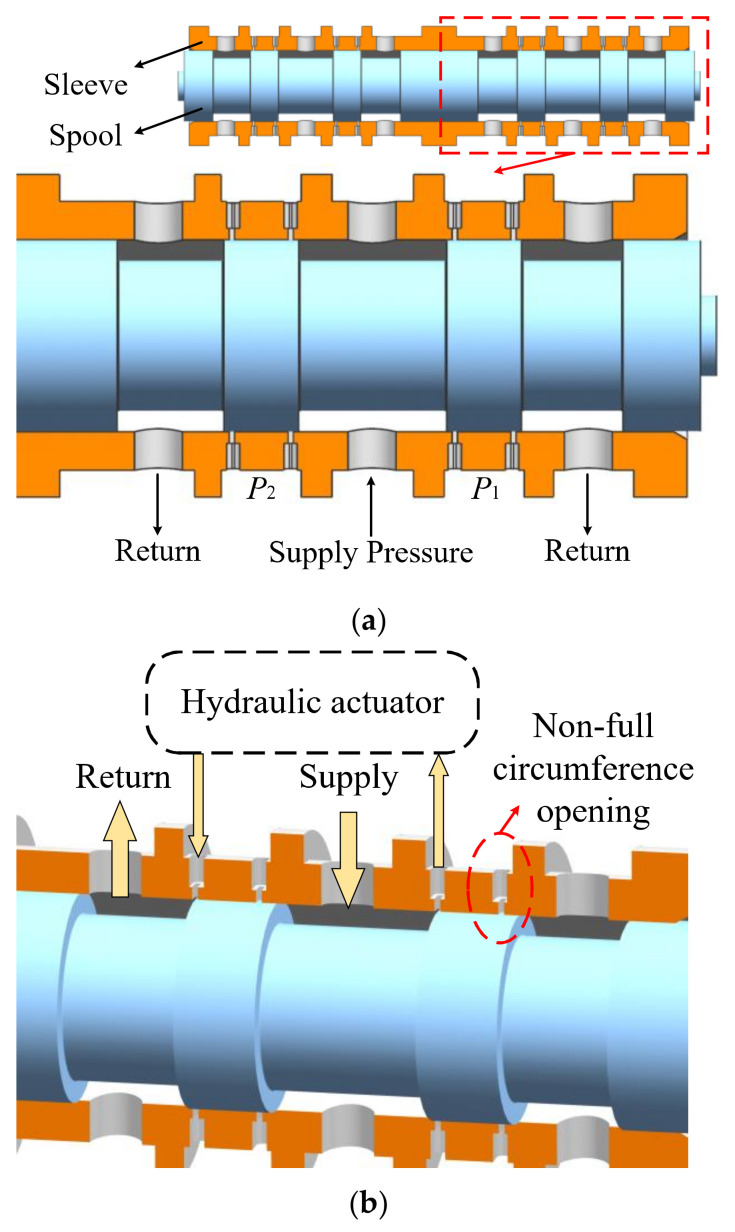
Geometry of direct drive servovalve: (**a**) Connection of two spools and geometry; (**b**) Flow in the valve chamber and valve openings.

The structure of the double-circuit and a single-circuit servovalve is shown in Figure 1a. This valve adopts non-full-circumference openings. Figure 1b shows the flow direction of each port at a specific opening and the geometry of openings. In general, the return pressure is as high as the atmospheric pressure, and the oil pressure from an actuator back to the servovalve is several megapascals. The pressure drop across the oil return cavity is large and is where cavitation tends to occur easily.

### 2.2. Simulation Approaches

**Figure 2:**
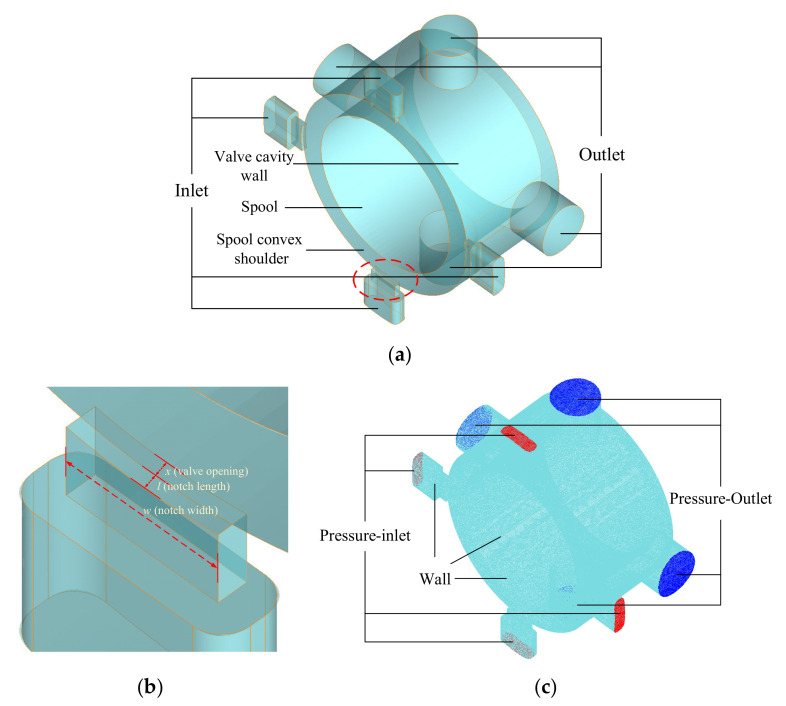
Flow domain for numerical calculation: (**a**) Flow field; (**b**) Geometry near the valve opening; (**c**) Meshing and boundary conditions.

The three-dimensional flow domain is shown in Figure 2a. The flow in the valve cavity can be considered as a steady-state. Meanwhile, with the throttling of valve openings, an unstable high-speed jet and cavitation exist mainly near valve openings. The liquid in the flow domain downstream far from the opening slows down significantly and tends to be stable. Therefore, the flow domain near valve openings is the focus of numerical calculation. In this paper, the mesh type is unstructured, and the mesh units are tetrahedral and hexahedral. The unstructured mesh is more suitable for dividing the flow field with complex shapes, and it is easy to control the grid size and node density. The resulting meshes are shown in Figure 2c.

To simplify the calculation, half of the double-circuit spool is modeled and numerically simulated, whose oil return cavity can reflect the relating behavior of cavitation and the induced oscillation. Details about the solver used can be referred to in Table 1. The continuity and momentum equations are solved by commercial software ANSYS Fluent 16.0. The governing equations and the equations of the Realizable k-ε turbulence model are proposed by Jun-ye Li et al. [24] Using the cavitation model proposed by Zwart et al. [25], the equations describing cavitation are written as follows:

(1) $$\frac{\partial }{\partial t}\left(\alpha \rho_{v}\right)+\nabla \left(\alpha \rho_{v}u_{v}\right)=R_{e}-R_{c}$$

(2) $$R_{e}=F_{vap}\frac{3\alpha_{nuc}\left(1-\alpha_{v}\right)\rho_{v}}{R_{B}}\sqrt{\frac{2}{3}\frac{p_{v}-p}{\rho_{1}}}\left(p\leq p_{v}\right)$$

(3) $$R_{c}=F_{cond}\frac{3\alpha_{v}\rho_{v}}{R_{B}}\sqrt{\frac{2}{3}\frac{p-p_{v}}{\rho_{1}}}\left(p\geq p_{v}\right)$$

where *α* is the vapor phase volume fraction, the subscript *ν* stands for the vapor phase, *ρ* stands for the fluid density, *μ* stands for the velocity, *R_e_* and *R_c_* are associated with the growth and collapse of bubbles, *R_B_*, *F_vap_*, and *F_cond_* are constant in the model. In this simulation, the SIMPLE (Semi-Implicit Method for Pressure Linked Equations) algorithm based on pressure and the second-order upwind discretization scheme were adopted.

### 2.3. Transient Cavitation Evaluation

The changes in the macroscopic morphology of cavitation include occurrence, movement, collapse, etc, and the transient cavitation will cause the changes of other parameters as well. In order to more accurately describe the severity of the evaporation phenomenon in the valve cavity, the local cavitation fraction *α_p_* and the surface average cavitation fraction *α_s_*, are defined [26,27].

For the mixture two-phase cavitation model, the cavitation situation is represented by vapor phase volume fraction *α*, and thus the volume fraction of the liquid phase is 1-*α* [28]. For transient flow with cavitation, the vapor phase volume fraction is a field function related to both position and time, which can be expressed as *α* (*x, t)*, where *x* represents the spatial position and *t* represents the time. When the position is fixed as *x_i_*, *α* (*x_i_, t)* can describe the local vapor volume fraction at point *i* [29].

**Figure 3:**
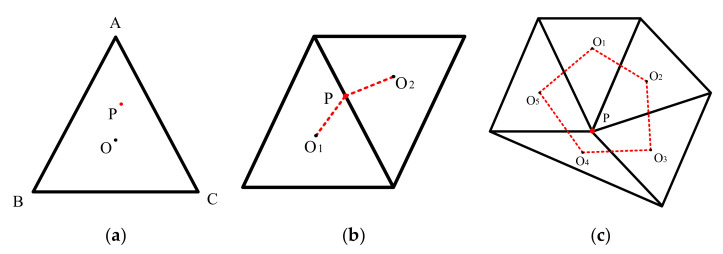
The situations in which the monitoring points are located in different locations: (**a**) Monitor point within a cell; (**b**) Monitor point on the edge of adjacent cells; (**c**) Monitor point at the node of adjacent cells.

The physical quantities in the flow field are stored in some discrete space points during the process of numerically solving. When calculating the cavitation fraction at a certain point P, it can be divided into two cases according to the position of P. First of all, when point P is located inside a computing unit, as shown in Figure 3a. It is considered that no matter where point P is located, as long as it is located inside the calculation cell, its cavitation fraction value is the vapor phase volume fraction value of point O, denoted as *α_p_* (*i, t*)*,* where *i* represents the sequence number of the cell in the whole mesh system. Secondly, when the selected point P is located at the boundary or node of adjacent computing units, as shown in Figure 2b,c. The cavitation fraction of point P is the algebraic average value of the cavitation fraction at the center of adjacent computing units, as shown in Equation (4), where *α_n_* represents the vapor phase volume fraction at the center of each computing unit adjacent to the boundary or node and *n* is the total number of adjacent cells.

(4) $$\alpha_{p}\left(t\right)=\frac{1}{N}\overset{N}{\underset{n=1}{\sum }}\alpha_{n}\left(i,t\right)$$

Because the local cavitation fraction only reflects the changes of the evaporation phenomenon at one point, the significance of it is limited in application. In order to quantify the cavitation on a surface, based on the local cavitation fraction *α_p_*, the surface average cavitation fraction *α_s_* is proposed. The surface average cavitation fraction *α_s_* is mainly used to describe the average cavitation effect on any surface, and its calculation method is shown in Equation (5), where *S* represents the total area of a surface. In the discrete domain of numerical calculation, the integral operation is transformed into a summation operation, as Equation (6), where *N* represents the number of all surface elements contained in the surface, and *α* (*i, t*) is the vapor volume fraction of the *i*-th surface element.

(5) $$\alpha_{s}\left(t\right)=\frac{1}{S}\underset{S}{\iint }\alpha \left(x,t\right)dS$$

(6) $$\alpha_{s}\left(t\right)=\frac{1}{S}\sum_{i=1}^{N}\alpha \left(i,t\right)∆S$$

**Figure 4:**
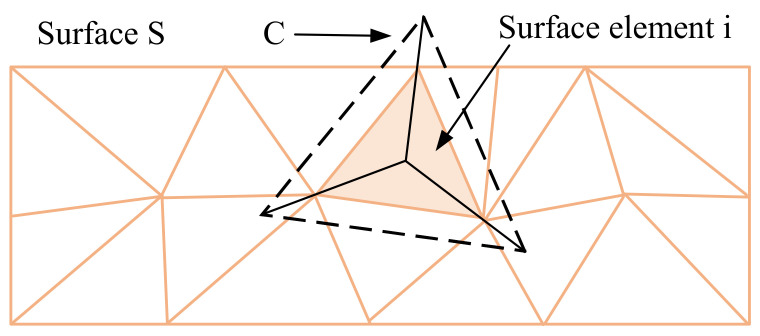
Calculation of discrete surface integral.

When calculating the surface average cavitation fraction *α_s_* (*t*) on the surface *S*, any surface element *i* on the surface is formed by the intersection of surface *S* and the calculation unit C, the principle of which is shown in Figure 4 [30]. Therefore, the cavitation fraction at the surface element *i* is determined by the value *α* at the center of the grid unit C to which the surface element belongs.

## 3. Results and Discussion

### 3.1. Cavitation in the Valve Cavity

If the local pressure is reduced to below the saturated vapor pressure. As a flow rate control component, throttling is the main working method of a servovalve. The oil pressure will drop greatly when flowing the narrow opening. Therefore, the oil vaporization in the valve cavity mainly occurs closest to the valve openings.

**Figure 5:**
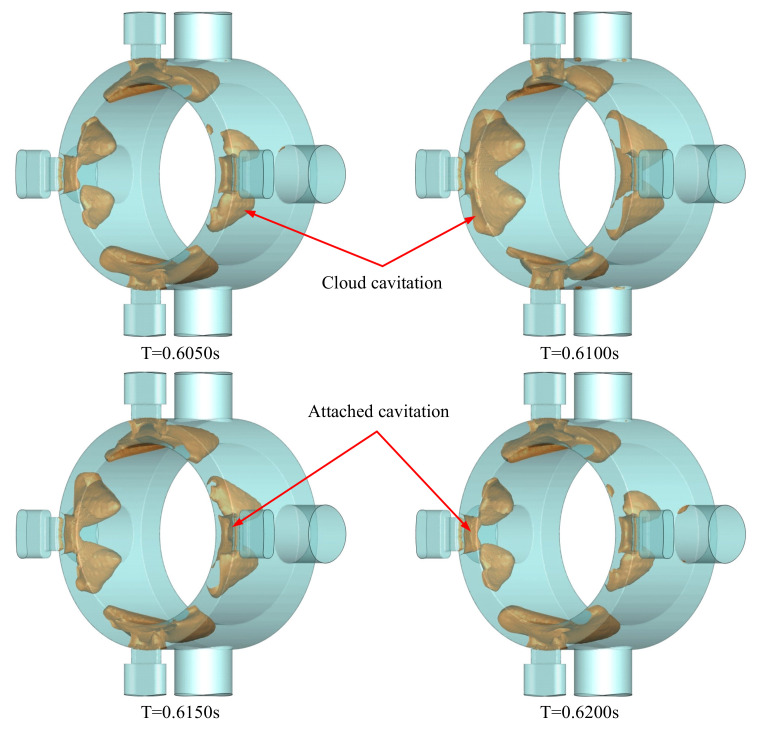
Morphological variation of cavitation in the valve cavity.

The time-varying bubbles in the valve cavity lead to a highly unstable cavitation configuration. Constant boundaries applied to the flow domain tend to visualize the cavitation from generation and evolution to collapse with a relatively certain regularity. The morphological variation of cavitation in the valve cavity is shown in Figure 5. Bubbles in the valve cavity are symmetrically distributed on both sides of the valve opening, as shown in Figure 5. The vapor volume fraction for the khaki-colored areas is higher than 0.2. The cavitation can be divided into two kinds: the attaching cavitation formed of bubbles close to the wall of the valve opening and the cloud cavitation formed of the shedding bubbles. The overall dynamics of the cavitation mainly depend on the nature of the latter. A cycle in which cavitation morphology changes is shown in Figure 5. At different times, the appearance of the attaching bubbles hardly changes, while the shedding bubbles along the direction of the jet flow exhibit a regular process of expansion and contraction. When the cavitation area composed of bubbles grows to be large enough, bubbles at the end of the attaching cavitation area gradually shed from the cavitation body. With shedding bubbles for the cloud cavitation, there is always a certain amount of attached cavitation at the valve wall surface. Finally, the cloud cavitation collapses and, as the fluid moves to a high-pressure area downstream, the attaching cavitation at the opening wall starts to grow again. The whole process goes round and begins again.

**Figure 6:**
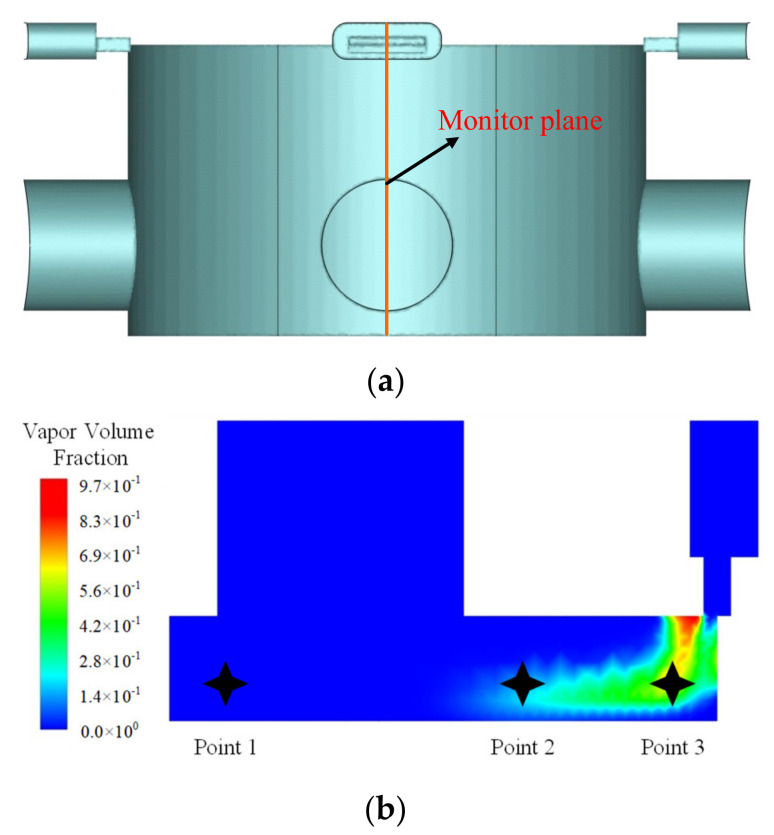
Location of the monitoring surface and points: (**a**) Monitoring surface; (**b**) Monitoring point.

The flow field configuration including the vapor fraction introduced above is time-variable. It is essential to analyze feature parameters of the cavitation and transient flow forces in the time domain and establish the related analysis method. The pulsation signals can be divided into stationary and non-stationary signals according to the variation law. Stationary signals are apt to remain their mean value for a long sampling duration. The mean value and pulsation amplitude were selected as two evaluation parameters in the time domain. Since the cavitation distribution is symmetrical on both sides of the valve openings, the axial cross-section of the spool was selected to monitor the cavitation. The monitoring surface and points are shown in Figure 6. Three locations are selected to monitor the cavitation as labeled in Figure 6b. Point 1 indicates the transition area where bubbles from the attaching cavitation shed to form the cloud cavitation. Point 2 indicates the end position of the cloud cavitation, and Point 3 indicates the other convex shoulder surface.

**Figure 7:**
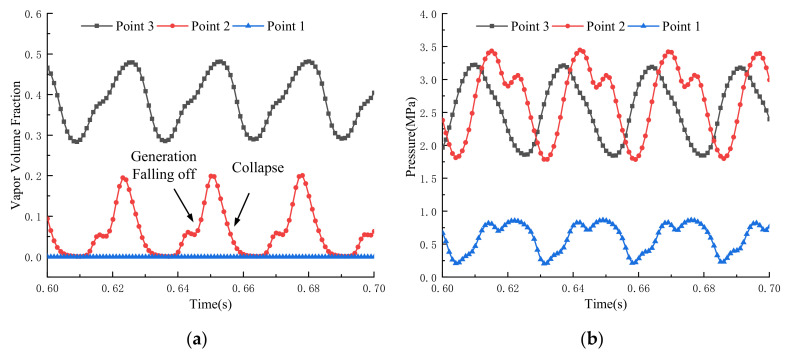
Fluctuations of local parameters at the monitoring points: (**a**) Local cavitation fraction; (**b**) Pressure.

The pulsation amplitude of pressure at Point 3 is the smallest, as shown in Figure 7. It is the smallest because Point 3 is comparatively far away from the cavitation area, and when the fluid moves close to Point 3, the bubbles have collapsed, and the pressure fluctuation transmits from the fluid upstream. The pressure pulsation frequency at Point 1 and Point 2 are almost identical. However, there exists a lag in the pressure pulsation at Point 2, which can attribute to the potential energy of bubbles in the corresponding cavitation area. At Point 1, the attaching cavitation transforms into the cloud cavitation and the pressure fluctuates at the earliest point there. Point 2, in contrast, is located at the end of cloud cavitation, and the pressure fluctuation relies on its energy in the cavitation with further evolution energy.

**Figure 8:**
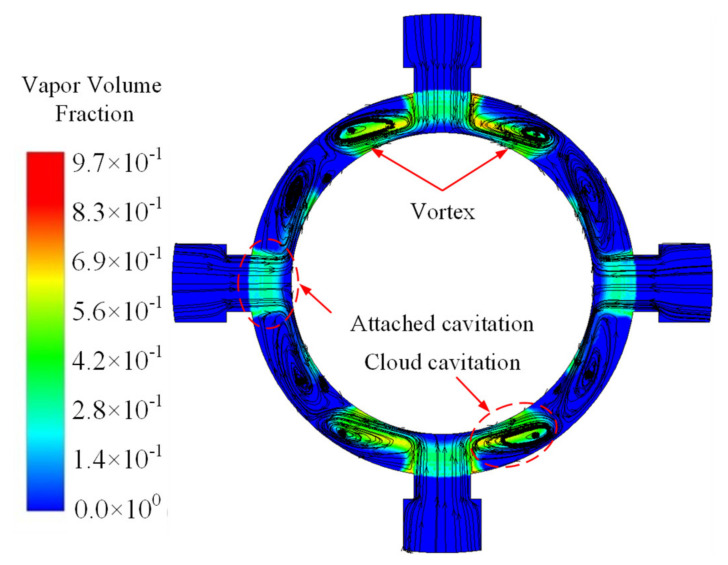
Streamline and cavitation on the section parallel to the convex shoulder surface.

**Figure 9:**
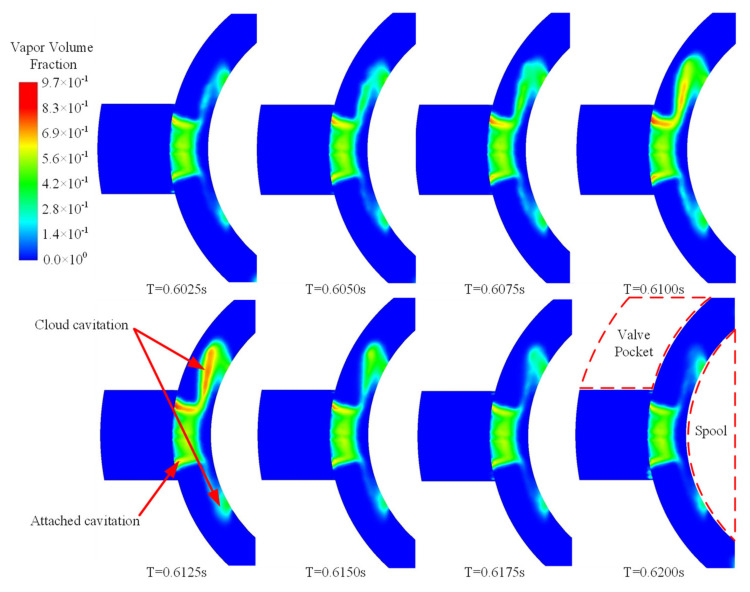
Morphological variation of cavitation on the convex shoulder surface with nearby openings.

A comparison between Figure 7a,b shows that the pressure pulsation is closely related to the shedding and movement of bubbles. The cloud cavitation will expand to other fields within the fluid domain with the jet flow after the bubbles shedding from the attaching cavitation. When the high-speed jet impinges on the spool or socket wall inside the valve, this is an obvious backflow, and the reverse jet is located on both sides of the forward jet. Therefore, the detaching cloud cavitation will appear on both sides of the high-speed jet, and eventually it will collapse, which induces the pressure with periodic pulsation at multiple positions. The distribution of streamlines and the cavitation in the section parallel to the convex shoulder surface is shown in Figure 8. When impacting on the valve stem surface, the high-speed jet bounces and induces vortices on both sides of the valve openings. The location of the vortex corresponds to the region of the cloud cavitation. The movement, diffusion, and collapse of the cloud cavitation can increase the instability of the flow field, and the pressure pulsation in the valve cavity will transmit to solid parts, such as a spool in the valve, when contacting with the solid wall. The cavitation distribution on the spool convex shoulder surface represents a strong time variation and periodicity, as shown in Figure 9.

**Figure 10:**
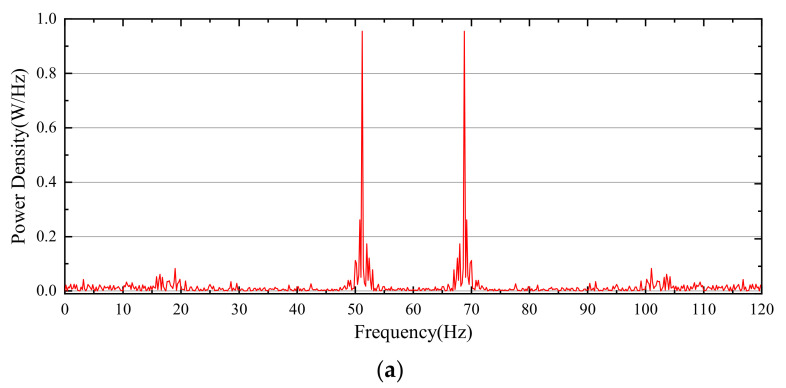

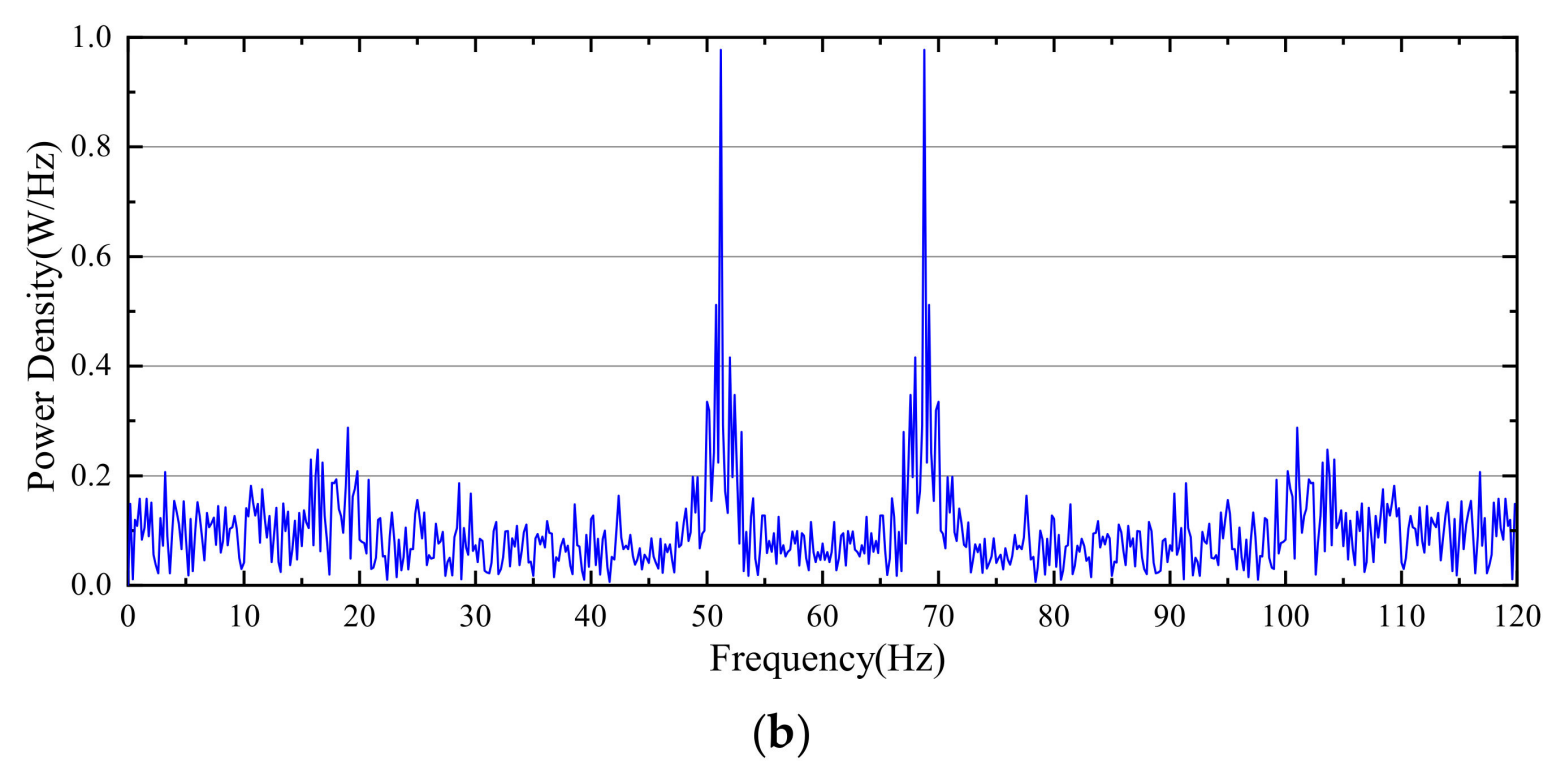
Frequency spectrum: (**a**) Surface average cavitation fraction; (**b**) Average pressure.

The average cavitation fraction and the average pressure on the specific surface are extracted and their frequency spectrum is shown in Figure 10. The spectrum distribution of the average cavitation fraction is relatively concentrated. The peak spectrum locates in the frequency band of 50 Hz and 70 Hz, and also there exists a spectrum around the frequency band of 100 Hz and 20 Hz. The spectrums at other frequency bands associate with the aggravated cavitation instability. Comparison between the two spectrums implies that the cavitation formation can induce pressure pulsation in the valve cavity, and increase the instability flow there, so as to generate pressure oscillation at more frequency bands. On the other side of the valve cavity, however, cavitation is negligible due to fluid with weak phase potential downstream in the domain (Figure 7). The fluctuation amplitude of the pressure is small, and its change is relatively gentle. The discrepancy in pressure pulsation frequency and amplitude on different convex shoulder surfaces of a spool triggers the spool oscillation. In addition, the oscillation is inclined to disturb cavitation occurrence and collapse, and even aggravate the flow stability further, which may lead to higher-frequency pressure pulsation.

### 3.2. Influencing Factor

**Figure 11:**
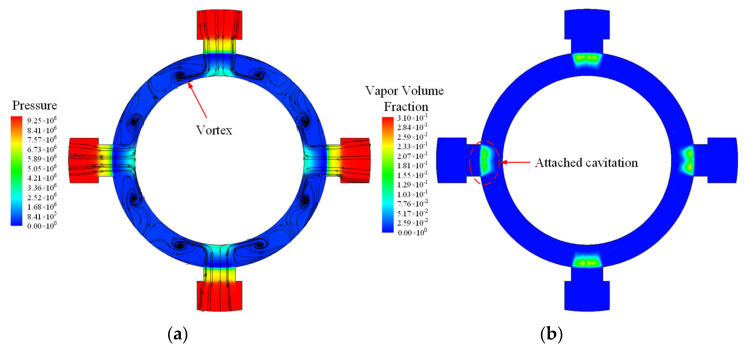
Demonstration with higher back pressure: (**a**) Pressure contour and streamlines; (**b**) Vapor phase contour.

To explore the influencing factor of the pressure pulsation and cavitation, backpressure with a 0.5 MPa increment was adopted for a practical method to reduce the oscillation caused by cavitation. A demonstration of this is shown in Figure 11. The pressure contour, streamlines, and vapor phase distribution contours indicate that the cavitation has been greatly reduced. The cloud cavitation seen in the original valve cavity no longer exists, and only the attaching cavitation exists at the valve openings. Meanwhile, the volume fraction of the vapor phase significantly decreases up to about 0.3. The vortices still exist in a smaller region. Figure 11a shows that jet flow impacting on the wall through the valve openings remain unchanged, but the pressure does not reduce to less than the saturated vapor pressure. In short, the bubbles’ volume and strength diminish considerably, which means that the cavitation in the valve cavity is greatly inhibited by increasing the backpressure.

**Figure 12:**
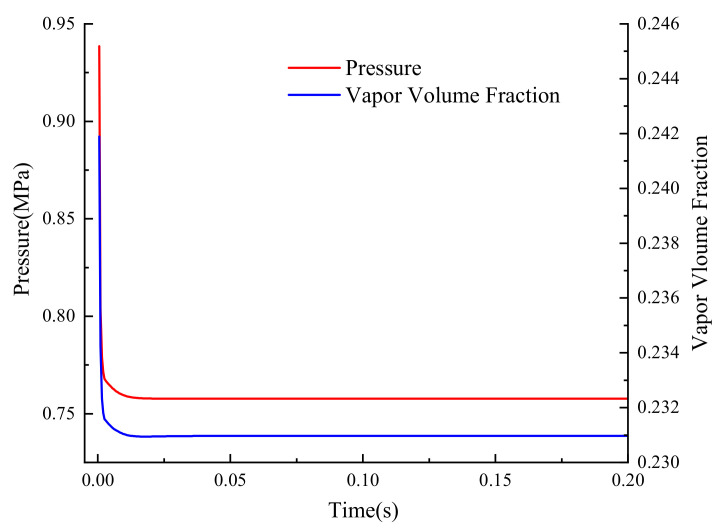
Local cavitation fraction and pressure.

A monitoring point in the attached cavitation near the valve openings was selected, where the pressure and vapor volume fraction are depicted in Figure 12. Both level off after a sudden change that was attributed to the modification of boundary conditions, and the fluctuations that existed before have disappeared. The results prove the relevance of the pressure and the cavitation. The spool oscillation caused by the pressure fluctuation has a clear mapping with the cavitation presented in this paper. The method of inhibiting the oscillation can be found by means of alleviating the cavitation in the valve cavity.

**Figure 13:**
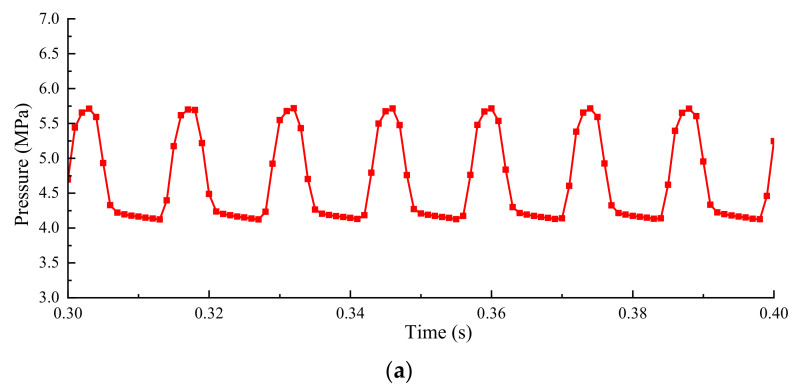

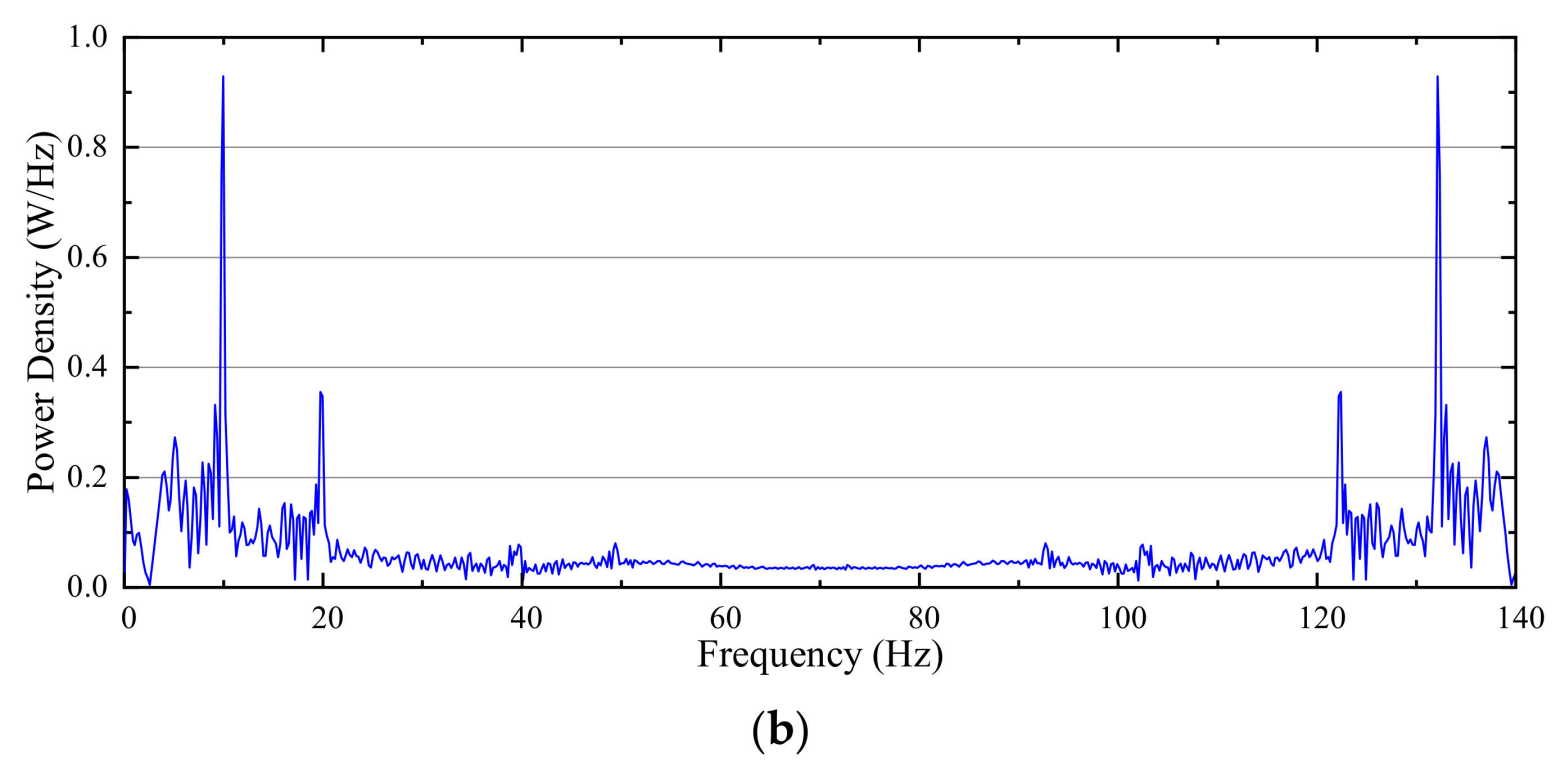
Pressure fluctuations of point 1 at inlet pressure 21 MPa: (**a**) Fluctuation in time domain; (**b**) Spectrum analysis.

When the inlet pressure is increased to 21 MPa and the outlet pressure is at a normal atmospheric pressure, the fluctuations of the pressure are shown in Figure 13, as compared to Figure 7. The pressure difference is larger at different sampling times and there are more cycles of pressure pulsation for the same length of time. What can be seen from the results of spectrum analysis is that the main frequency band is distributed around 130 Hz and 10 Hz, which means a higher frequency of pressure pulsation in the valve cavity. In addition, the amplitude of pressure pulsation also increased to 1.5 MPa, which causes the spool to be subjected to higher energy pressure shocks. The results reflect the synchronization of the pressure pulsation and cavitation again. Meanwhile, the spool oscillation caused by pressure pulsation has an indelible relationship with cavitation. The method of inhibiting the oscillation of spools can be considered from the direction of relieving cavitation in the valve cavity.

## 4. Conclusions

Here we have investigated spool oscillation in a new double-circuit direct drive servovalve using numerical simulation. Our experimental approach reveals the growing process of bubbles causing the cavitation which affects the spool oscillation.

(1) The oil return valve cavity is the major region of bubble concentration due to its cavitation.
(2) The average cavitation fraction was evaluated at selected monitoring positions. The numerical results indicate that the bubbles in the valve cavity greatly change, although the growth, evolution, and collapse stages are cyclical. Analysis of the pressure and the local cavitation volume fraction at each monitoring point suggests a strong correlation between the pressure fluctuation and the amount of cavitation.
(3) A spectrum analysis of the average cavitation fraction and the average pressure on the surface of the convex shoulder were both almost identical. This suggests that the leading cause of cavitation is the pressure pulsation. If we increase the pressure at the return port of the valve, this can inhibit oscillation. Whereas, the amplitude and frequency of pressure pulsation are boosted with an increase in inlet pressure.

In summary, the oscillation of movable elements inside hydraulic components with a large pressure drop across an opening or restrictor is highly related to bubbles that create cavitation. Further research is recommended to discover more effective approaches to reduce the amount of bubbles and cavitation to help increase the reliability and output power of servovalves.

## Figures and Tables

**Table 1:** Solver and Settings for the simulation.

**Solution Approaches**	
Solver	SIMPLE algorithm
Turbulent model	Large-eddy simulation
Subgrid-Scale model	WALE
Multi-phase model	Mixture
Cavitation model	Schner-Sauer
Discretization of the pressure equation	PRESTO!
Discretization of momentum equation	Second-order upwind
Discretization of volume fraction	First-order upwind
Under-relaxation of pressure	0.75
Under-relaxation of momentum	0.75
Under-relaxation of volume fraction	0.75
**Boundaries and Fluid Parameters**	
Time step size	1.00 × 10^−5^
Inlet pressure	10.0 MPa
Outlet pressure	Atmospheric pressure
Liquid oil density	889 kg/m^3^
Liquid oil viscosity	0.045 kg/(m s)
Vaporized oil density	0.55 kg/m^3^
Vaporized oil viscosity	1.34 × 10^−5^ kg/(m s)
Vaporized pressure	10 Pa
Bubble number density	1.00 × 10^13^
